# Limited Utility of Routine Tests Prior to Ophthalmologic Surgery: An Observational Study in a Japanese Hospital

**DOI:** 10.31662/jmaj.2020-0112

**Published:** 2021-07-06

**Authors:** Mitsuhiro Matsuo, Yoshinori Takemura, Mitsuaki Yamazaki

**Affiliations:** 1 Department of Anesthesiology, Faculty of Medicine, University of Toyama, Toyama, Japan

**Keywords:** Choosing Wisely campaign, routine diagnostic tests, general anesthesia, low-risk surgery, postoperative complications

## Abstract

**Introduction:**

Routine preoperative testing for low-risk surgeries without a clinical indication should be avoided; however, such tests are still frequently performed in Japan. This study was performed to assess the impact of routine preoperative tests in low-risk surgery in a Japanese medical setting.

**Methods:**

We performed a retrospective chart review to examine the utility of routine tests with respect to anesthetic management and postoperative complications in all patients aged ≥ 18 years whom ophthalmologists consulted with anesthesiologists before ophthalmologic surgery under general anesthesia.

**Results:**

During the 10-year study period, 1,234 anesthetic consultations and 1,211 routine preoperative tests (laboratory tests, chest X-rays, and electrocardiograms) were performed in Toyama University Hospital. In total, 59 patients (4.8% of the study population) canceled surgery after a battery of preoperative evaluation. Among them, 10 patients had incidental abnormalities that necessitated additional tests, and only three patients (0.2%) canceled surgery. In-hospital postoperative complications developed in nine patients (0.7%) whose routine test results made it difficult to predict development of these adverse events. No severe life-threatening events were noted in this survey.

**Conclusions:**

Routine tests prior to eye surgery for adults were of low value for perioperative management and prediction of development of in-hospital complications in this Japanese medical setting. Anesthesiologists and ophthalmologists should selectively order preoperative tests based on the medical interview and physical examination.

## Introduction

Preoperative examinations such as laboratory tests, chest X-rays (CXRs), and electrocardiograms (ECGs) are often necessary and can be invaluable for diagnosis, assessment, and treatment ^(1)^. In contrast, routine testing is discouraged before low-risk surgery because such testing is of low value and provides no beneficial effects in terms of patient outcomes ^(2)^. Severe adverse events associated with cataract surgery are rare ^(3)^, and a systematic review comparing routine preoperative testing with either no testing or selective testing showed that routine preoperative testing increased surgical costs but did not reduce the risk of perioperative adverse events ^(4)^. Several professional societies, including the American Society of Anesthesiologists (ASA), state that routine preoperative testing should be avoided in patients undergoing low-risk surgeries without a clinical indication as part of the Choosing Wisely campaign, which encourages healthcare providers and professional societies to lead efforts to reduce the use of low-value care^(1), (5)^. The National Institute for Health and Care Excellence guideline also states that routine preoperative tests should be avoided in patients with an ASA physical status of 1 or 2 undergoing minor surgery ^(6)^. However, preoperative blood tests are still frequently performed even before low-risk surgeries in some countries, including Japan^(7), (8)^.

In this study, to assess the impact of routine preoperative tests in patients undergoing low-risk surgery in a Japanese medical setting, we conducted a retrospective chart review to examine the utility of routine tests with respect to anesthetic management and postoperative complications in patients undergoing ophthalmologic surgery under general anesthesia.

## Materials and Methods

### Study design

This retrospective cohort study was approved by the ethics committee of Toyama University Hospital (No. R2020129) and conducted in accordance with the principles of the Declaration of Helsinki. We identified all patients aged ≥ 18 years for whom ophthalmologists consulted with anesthesiologists before eye surgery in Toyama University Hospital from January 2010 to December 2019 using electronic medical records. Incidental abnormalities found in routine tests were defined as abnormalities that have been difficult to predict based on the patients’ medical history and physical examination findings by attending anesthesiologists. Routine laboratory blood tests included a complete blood count, liver function tests (aspartate aminotransferase, alanine aminotransferase, alkaline phosphatase, total bilirubin, and albumin), kidney function tests (creatinine with or without estimated glomerular filtration rate), glucose metabolism (random blood glucose with or without hemoglobin A1c [HbA1c]), and electrolytes (sodium, potassium, and chloride). Abnormalities in CXR and ECG were assessed by the attending anesthesiologists. The purpose of this study is to investigate the effect of routine preoperative tests on anesthetic management and postoperative systemic complications during hospitalization. Complications were defined and classified according to the Clavien-Dindo classification as any deviation in the normal postoperative course ^(9)^.

### Statistical analysis

Continuous variables are presented as median (interquartile range [IQR]), and categorical variables are presented as number (percent) of patients in each group. The Mann-Whitney test was used to evaluate statistical significance. A *P* value of <0.05 was considered statistically significant.

## Results

**Figure 1.**
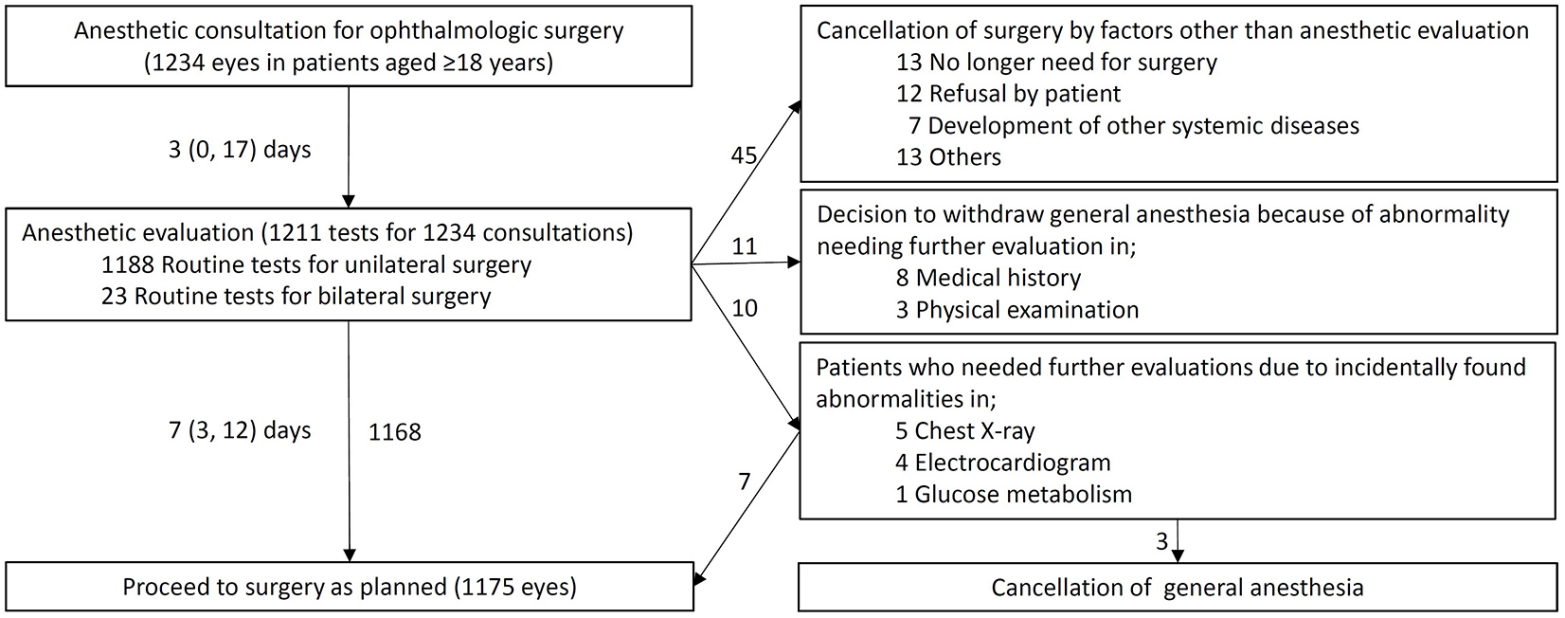
Timeline of a battery of preoperative evaluation before eye surgery in our hospital. Interquartile range is shown in parentheses.

Preoperative laboratory screening, CXR, and ECG were routinely performed before anesthetic evaluation in our hospital (Figure 1). During the 10-year study period, 1,211 routine preoperative tests were performed at a mean of 7 (3-12) days before surgery: 1,188 for unilateral eye surgeries and 23 for bilateral eye surgeries. As a result of anesthetic evaluation, 1,175 consultations (95.2%) proceeded to surgery as planned.

The characteristics of patients who underwent surgery (n = 1,175) are summarized in Table 1. The patients’ median age was 66 years, 605 were male (51%), and 1,169 were Japanese (99%). The most frequent comorbidities were hypertension (36.2%) and diabetes mellitus (16.3%), followed by psychosis (10.4%) and dementia (10.4%). Surgical procedures included vitrectomy (22.5%), cataract (22.1%), strabismus (20.9%), transplantation (9.7%), and trauma (7.9%). The median operative time was 68 (43-107) min, and the bleeding volume was too small to measure in almost all surgeries.

**Table 1.** Characteristics of Patients Who Underwent Ophthalmologic Surgery under General Anesthesia for 10 Years.

	Total (n = 1175)
Age, years (IQR)	66 (43-77)
Male (%)	605 (51)
Japanese (%)	1169 (99)
Body mass index, kg/m^2^ (IQR)	23 (20-25)
Comorbidities (%)	
Hypertension	425 (36.2)
Diabetes mellitus	192 (16.3)
Psychosis	122 (10.4)
Dementia	122 (10.4)
Neurologic disease	97 (8.3)
Intellectual disability	88 (7.5)
History of stroke	88 (7.5)
Arrhythmia	68 (5.8)
Lung disease	56 (4.8)
History of heart failure	12 (1.0)
End-stage renal disease	9 (0.8)
ASA classification (%)	
1 or 2	999 (85)
3 or 4	176 (15)
Surgical procedure/Indication (%)	
Vitrectomy	264 (22.5)
Cataract	260 (22.1)
Strabismus	246 (20.9)
Transplantation	114 (9.7)
Trauma	93 (7.9)
Others	198 (16.9)
Emergency (%)	67 (5.7)
Operative time, min (IQR)	68 (43-107)
Bleeding volume, mL (IQR)	0 (0-0)

Continuous variables are presented as median (interquartile range [IQR]), and categorical variables are presented as number (percent) of patients in each group. ASA, American Society of Anesthesiologists.

In all patients, any out-of-range values in the complete blood count, liver function tests, kidney function tests, electrolytes, or glucose were found in 513 (42%), 641 (53%), 213 (18%), 261 (22%), and 518 (43%) patients, respectively. Any abnormalities in CXR and ECG were identified in 181 (15%) and 338 (28%) patients, respectively. As shown in Table 2, 59 patients (4.8% of the study population) canceled surgery after a battery of preoperative evaluation. Among them, 10 patients had incidental abnormalities that necessitated additional tests after anesthetic evaluation, and only three patients canceled surgery. Bronchiectasis localized to the right lung was identified in the CXR of a 78-year-old man who was in good condition to perform high-intensity work such as snow shoveling (estimated as ≥ 4 metabolic equivalents) ^(10)^. After consultation with the pulmonologist, the specialist instructed us to cancel his cataract surgery. An 85-year-old woman with a history of hypertension and cerebral infarction was newly diagnosed with atrial fibrillation. Three months after a visit with a cardiologist, she underwent cataract surgery with the use of an oral anticoagulant. In an asymptomatic woman aged 73 years, the HbA1c concentration was incidentally found to be 9.3%. After 2 months of glycemic control, her HbA1c decreased to 7.2%, and she proceeded to surgery with another anesthetic evaluation. Although other 23 patients with diabetes (1.9%) also had an HbA1c concentration of ≥ 8.5% in this survey, all these patients proceeded to undergo surgery as planned.

**Table 2.** Patients Who Required Further Evaluations because of Incidentally Found Abnormalities in Routine Tests.

	Patient		Abnormalities in routine tests	Further evaluations	Surgery under general anesthesia
Chest X-ray					
	78	M	Bronchiectasis	Chest CT, pulmonologist	Canceled
	71	M	Cardiomegaly	TTE, cardiologist	Proceed as planned
	78	M	Cardiomegaly	TTE, cardiologist	Proceed as planned
	82	F	Possible atelectasis	Chest CT	Proceed as planned
	86	F	Widening of the aortic silhouette	Chest CT	Proceed as planned
Electrocardiogram					
	85	F	Atrial fibrillation	TTE, cardiologist	Canceled
	79	M	Abnormal Q wave	TTE, cardiologist	Proceed as planned
	85	F	Non-specific ST elevation	TTE, cardiologist	Proceed as planned
	69	F	Right axis deviation	TTE, cardiologist	Proceed as planned
Glucose metabolism					
	73	F	Hemoglobin A1c 9.3	Diabetologist	Canceled

CT, computed tomography; TTE, transthoracic echocardiography.

As shown in Table 3, in-hospital postoperative complications were noted in nine patients (0.7%). A 78-year-old man with a body mass index of 20.5 kg/m^2^ developed tachypnea and hypoxemia immediately after extubation and was diagnosed with negative-pressure pulmonary edema. Postoperative nausea and vomiting developed in two nonsmoking patients. A 72-year-old man who developed transient respiratory depression due to residual neuromuscular blockade had normal liver and kidney function test results preoperatively. The preoperative ECG findings were not significantly abnormal in a 77-year-old woman who developed chest discomfort immediately after arousal from general anesthesia. Her cardiologists denied new-onset myocardial ischemia and transferred the patient to her primary care physician for further observation. An 81-year-old man who developed transient grade 1 acute kidney injury had a preoperative serum creatinine concentration of 0.92 mg/dL (estimated glomerular filtration rate, 60.2 mL/min/1.73 m^2^) ^(11)^. A 70-year-old man who was undergoing treatment with warfarin and had a preoperative international normalized ratio of 2.49 developed nasal bleeding after dacryocystorhinostomy, and he required embolotherapy by radiologists on postoperative day 6. None of the seven above-described patients with abnormal results of routine tests had postoperative complications. There was no significant difference in the length of the postoperative hospital stay between patients with complications (5 days; IQR, 1-6 days) and those without complications (2 days; IQR, 1-5 days), although postoperative complications developed in only nine patients.

**Table 3.** In-Hospital Complications Developed after Ophthalmologic Surgery.

Patient		Postoperative complication	Comorbidity	Length of postoperative stay, day
72	M	Residual neuromuscular blockade	Hypertension	1
77	F	Chest discomfort, ECG change	Hypertension	1
45	M	Postoperative delirium	Psychosis	1
67	M	PONV	Hypertension	2
78	M	Negative pressure pulmonary edema	Peripheral arterial disease	5
77	F	PONV	Hypertension, diabetes mellitus	6
81	M	Acute kidney injury	Diabetes mellitus, internal carotid artery stenosis	6
70	M	Nasal bleeding	Atrial fibrillation on warfarin	27
76	F	Exacerbation of psychosis	Psychosis, arrhythmia requiring pacemaker implantation	107

ECG, electrocardiogram; PONV, postoperative nausea and vomiting

## Discussion

We performed this retrospective study to assess the impact of routine tests before ophthalmologic surgery on anesthetic management and postoperative complications. Only three of 10 patients (0.2% of the study population) who needed additional tests following incidentally found abnormalities in the routine preoperative tests canceled surgery. The remaining seven patients proceeded to undergo surgery after further evaluations, indicating that their further evaluations had been excessive and time-consuming ^(12)^. Nine patients developed in-hospital postoperative complications that would have been difficult to predict based on the results of the routine tests.

The choice of anesthetic technique and management would not be affected by the results of routine tests prior to ophthalmologic surgery. Typically, general anesthesia is selected for adults who are unable to communicate, cooperate, or remain stationary during eye surgery. The American College of Cardiology/American Heart Association guideline recommends that asymptomatic patients with a functional capacity of ≥ 4 metabolic equivalents proceed with their planned surgery ^(13)^. The most reliable tests for predicting difficult airways are physical examinations, such as the modified Mallampati test and the upper lip bite test ^(14)^. The preoperative hemoglobin level does not seem important because the bleeding volume during ophthalmologic surgery is too small to necessitate blood transfusion (Table 1). No strict dosage adjustments are necessary in patients with renal or hepatic failure who undergo surgery with frequently used anesthetics such as remifentanil and propofol ^(15)^. Additionally, the operative time is generally not long enough to require strict fluid management ^(16)^. Although there is a lack of consensus that antiplatelets and warfarin should be continued for patients undergoing eye surgery other than cataract ^(17)^, there is a possibility that coagulation abnormalities have minimal impact on vitrectomy ^(18)^.

For patients undergoing moderate- or high-risk surgery, routine preoperative tests contribute to the prediction of postoperative complications ^(19)^. Regarding ophthalmologic surgeries, the risk of postoperative complications varies between cataract and more invasive surgery, such as strabismus ^(20), (21)^. In this study, postoperative complications developed in only nine patients (0.8%), among whom moderate complications, such as pulmonary edema and acute kidney injury, were noted. Patients with a predisposition to upper airway obstruction, including patients with obesity, are at the greatest risk for negative-pressure pulmonary edema; this complication occurs after 0.05% to 0.10% of all procedures involving intubation and general anesthesia^(22), (23)^. The risk factors for postoperative acute kidney injury are a high body mass index, advanced age, and perioperative sepsis in major surgery ^(24)^. The commonly used risk score for postoperative nausea and vomiting created by Apfel and colleagues ^(25)^ is mainly calculated using the results of the medical interview, not the laboratory tests. These data suggest that routine tests prior to ophthalmologic surgery, such as cataract and vitrectomy, are unhelpful for the prediction of postoperative complications.

Our study raises questions about whether reducing the performance of routine preoperative tests is feasible in the healthcare system in Japan. One study in the United States showed that a multipronged intervention was associated with a marked and sustained decline in low-value preoperative testing for patients undergoing cataract surgery ^(26)^. Of interest, the authors extrapolated income losses from the perspective of the fee-for-service payment health system and extrapolated saving from the perspective of the society. In the fee-for-service system for outpatient care in Japan, the environment would be a potential barrier to eliminating low-value care.

A major limitation of this study is the definition of postoperative complications. Postoperative complications, such as myocardial injury and pneumonia, are often defined as adverse events that occur ≤ 30 days after surgery ^(27), (28)^. From an anesthetic viewpoint, we counted systemic or life-threatening events as postoperative complications, but we did not count ophthalmologic problems, such as local infection. Additionally, the characteristics of our patients may differ from those of Japanese adults in the general population. The incidence of diabetes in our study population was higher than expected ^(29)^, possibly because the surgical indications included vitrectomy for diabetic retinopathy. Notably, substantial numbers of patients with psychosis, dementia, and intellectual disability that were included in this survey possibly due to general anesthesia is selected for adults who are unable to remain immobile during eye surgery. Further studies are needed to assess the utility of routine preoperative tests in low-risk surgeries other than eye surgery.

In conclusion, routine tests prior to eye surgery for adults were of low value for perioperative management and prediction of the development of in-hospital complications in this Japanese medical setting. Anesthesiologists and ophthalmologists should selectively order preoperative tests based on the medical interview and physical examination.

## Article Information

### Conflicts of Interest

None

### Acknowledgement

We thank Mr. Toshio Fujimori for collecting clinical data and Angela Morben, DVM, ELS, from Edanz Group (https://en-author-services.edanzgroup.com/ac), for editing a draft of this manuscript.

### Author Contributions

All authors contributed to the study conception and design. MM performed the data collection and analysis, and YT and MY supervised the manuscript preparation. All authors read and approved the final manuscript.

### Informed Consent

As this was a retrospective study, consent for publication was not obtained from the participants.

### Approval by Institutional Review Board (IRB)

This retrospective cohort study was approved by the ethics committee of Toyama University Hospital (No. R2020129) and conducted in accordance with the principles of the Declaration of Helsinki.
